# Use of benzodiazepine receptor agonists in different pregnancy trimesters and risk of maternal and neonatal outcomes: a propensity weighted cohort study in Taiwan

**DOI:** 10.1186/s12884-025-08549-1

**Published:** 2025-12-06

**Authors:** Ruei-Siang Yu, Sheng-Yin To, Yi-Lin Chiang, Yu-Chieh Huang, Ke-Ting Pan, Hui-Wen Yang, Liang-Hsuan Chen, Li-Ting Kao, Ming-Fa Hsieh

**Affiliations:** 1 Department of Biomedical Engineering, Chung Yuan Christian University, No. 200, Zhongbei Rd., Zhongli Dist, Taoyuan, 320314 Taiwan; 2 Department of Clinical Pharmacy, Hualien Armed Forces General Hospital, Hualien, Taiwan; 3 School of Pharmacy, College of Pharmacy, National Defense Medical University, No.161, Sec. 6, Minquan E. Rd., Neihu Dist, Taipei, 114201 Taiwan; 4 Graduate Institute of Life Sciences, College of Biomedical Sciences, National Defense Medical University, Taipei, Taiwan; 5 School of Public Health, College of Public Health, National Defense Medical University, Taipei, Taiwan; 6 Department of Psychiatry, Tri-Service General Hospital, Taipei, Taiwan; 7 Center for Gender and Health Studies, National Defense Medical University, Taipei, Taiwan; 8 Graduate Institute of Aerospace and Undersea Medicine, College of Biomedical Sciences, National Defense Medical University, Taipei, Taiwan; 9 Department of Obstetrics and Gynecology, Chang Gung Memorial Hospital Linkou Medical Center, Taoyuan, Taiwan; 10 Department of Pharmacy Practice, Tri-Service General Hospital, Taipei, Taiwan

**Keywords:** Benzodiazepine receptor agonists, Pregnancy, Insomnia, Stillbirth, Trimester, Congenital malformations

## Abstract

**Background:**

The association between benzodiazepine receptor agonist (BZRA) exposure and adverse perinatal outcomes remains inconclusive. This study aimed to assess the risks of adverse pregnancy and neonatal outcomes associated with maternal BZRA exposure across pregnancy trimesters.

**Methods:**

Data from 170,144 maternal BZRA users and 1,098,172 nonusers were obtained from the Taiwan’s National Health Insurance database. We used cross-sectional study design with inverse probability of treatment weighting (IPTW) to evaluate the association between maternal BZRA exposure during each trimester and adverse pregnancy outcomes. The pregnancy period was classified into 4 intervals: preconception, first trimester, second trimester, and third trimester. Logistic regression was performed to estimate odds ratios (ORs) and 95% confidence intervals (CIs).

**Results:**

Maternal BZRA exposure was associated with an increased risk of stillbirth (IPTW-OR 1.19, 95% CI 1.14–1.25), preterm birth (IPTW-OR:1.11, 95% CI 1.09–1.13), low birth weight (IPTW-OR:1.05, 95% CI 1.03–1.07), Apgar score < 7 (IPTW-OR:1.17, 95% CI 1.12–1.22), and cesarean delivery (IPTW-OR:1.15, 95% CI 1.14–1.17) compared with nonusers. By timing, preconception exposure was modestly associated with preterm birth and cesarean delivery, and first-trimester exposure showed similar associations. The associations were most pronounced in the second trimester, with significantly elevated risks of stillbirth (2.29, 95% CI 2.11–2.48), preterm birth (1.38, 95% CI 1.33–1.43), Apgar score < 7 (2.05, 95% CI 1.92–2.20), low birth weight (1.35, 95% CI 1.29–1.40), small for gestational age (1.07, 95% CI 1.03–1.11), cesarean Sect. (1.21, 95% CI 1.18–1.24), and overall congenital malformations (1.27, 95% CI 1.13–1.43).

**Conclusions:**

This study observed a small but statistical association between BZRA exposure and several adverse pregnancy outcomes, which extended from the pre-pregnancy period through all trimesters. Clinicians should carefully weigh the risks and benefits when treating women who are pregnant or planning pregnancy.

**Supplementary Information:**

The online version contains supplementary material available at 10.1186/s12884-025-08549-1.

## Introduction

Pregnancy is a major life event in women that may contribute to psychological changes and worsen their original mental illness [1, 2]. Most researchers have recognized that untreated maternal psychiatric disorders may lead to adverse pregnancy outcomes and enduring detrimental effects for mothers, infants, and families [3–5]. Nevertheless, exposure to several psychotropic medications has been associated with adverse perinatal outcomes, such as miscarriage, preterm delivery, and low birth weights (LBW) [6, 7]. Therefore, there exists a consensus that the psychopharmacological treatments administered during pregnancy are uncertain and challenging to both patients and physicians.

Benzodiazepine receptor agonists (BZRAs), including benzodiazepines and Z-drugs, bind to the inhibitory neurotransmitter gamma-aminobutyric acid receptors in the central nervous system [8]. Clinically, BZRAs are primarily prescribed for depression, anxiety, and insomnia [9]. Because these sedative–anxiolytic agents were developed to provide a more favorable safety profile than barbiturates, particularly with lower risks of respiratory depression and dependence, they have become among the most widely prescribed medications [10]. However, the safety of BZRA prescription for pregnant women, especially in different trimesters, still remain under discussion. A recent study reported that the global prescription rate of BZRAs was approximately 1.9%, with the highest prevalence being found in the third trimester [11]. In Taiwan, approximately 3.4% of pregnant women had received BZRAs, with the highest incidence rate in the first trimester [12]. Because BZRAs and their metabolites pass through the placental barrier and accumulate in the fetal circulation [13–15], their use during pregnancy may affect fetal development and increase the risk of congenital malformations [13, 16, 17]. Two population-based studies have reported an elevated risk of congenital anomalies among mothers who used BZRAs during the first trimester [18, 19]. However, most previous investigations have focused solely on first-trimester exposure or on congenital malformations as outcomes, even though the fetus remains vulnerable to medication effects throughout all stages of in utero development. The first trimester is the critical period for organogenesis, whereas the second and third trimesters are essential for organ differentiation and rapid fetal growth [17, 20, 21]. Furthermore, a recent meta-analysis found that antenatal exposure to benzodiazepines was associated not only with congenital malformations but also with an increased risk of preterm birth, LBW, and small for gestational age (SGA) infants [22]. Nevertheless, evidence remains limited regarding how BZRA exposure during different trimesters, including the preconception period, relates to a broader spectrum of adverse pregnancy outcomes.

Large-scale epidemiological studies that systematically examine the effects of intrauterine BZRA exposure across different trimesters and its impact on obstetric and neonatal outcomes are still lacking. Therefore, this study aimed to comprehensively investigate the risks of adverse maternal and neonatal outcomes associated with maternal exposure to BZRAs during different pregnancy trimesters.

## Methods

### Data collection

Data were collected from the National Health Insurance (NHI) database and Birth Certificate Application maintained by the Health and Welfare Data Science Center, Ministry of Health and Welfare, Taiwan. The NHI, which started in 1995, covers > 99.9% of 23 million Taiwanese citizens and provides comprehensive medical interventions and services for all insured Taiwanese residents. Accordingly, the NHI claims data include the demographic, inpatient, and outpatient records that could track the medication prescription and diagnosis for every pregnant woman in Taiwan. The Birth Certificate Application captures all live births and stillbirths of ≥ 20 weeks’ gestation or birth weight ≥ 500 g, and includes information on infant birth date, gestational age, birth weight, sex, and congenital malformations diagnosed at delivery or within 7 days postpartum. Because medical institutions are legally required to register every birth within this period, the data are considered highly complete and accurate. This study linked the NHI database with Birth Certificate Application for further analysis using unique personal identification numbers. All personal identifiers were encrypted before being released to the researchers. This study was approved by the Institutional Review Board of the Taiwan Tri-Services General Hospital (TSGHIRB C202105085), and the requirement for informed consent was waived by the Institutional Review Board because all data were anonymized and de-identified. The Strengthening the Reporting of Observational Studies in Epidemiology (STROBE) reporting guideline was followed.

### Study sample selection and BZRA exposure definition

**Fig. 1:**
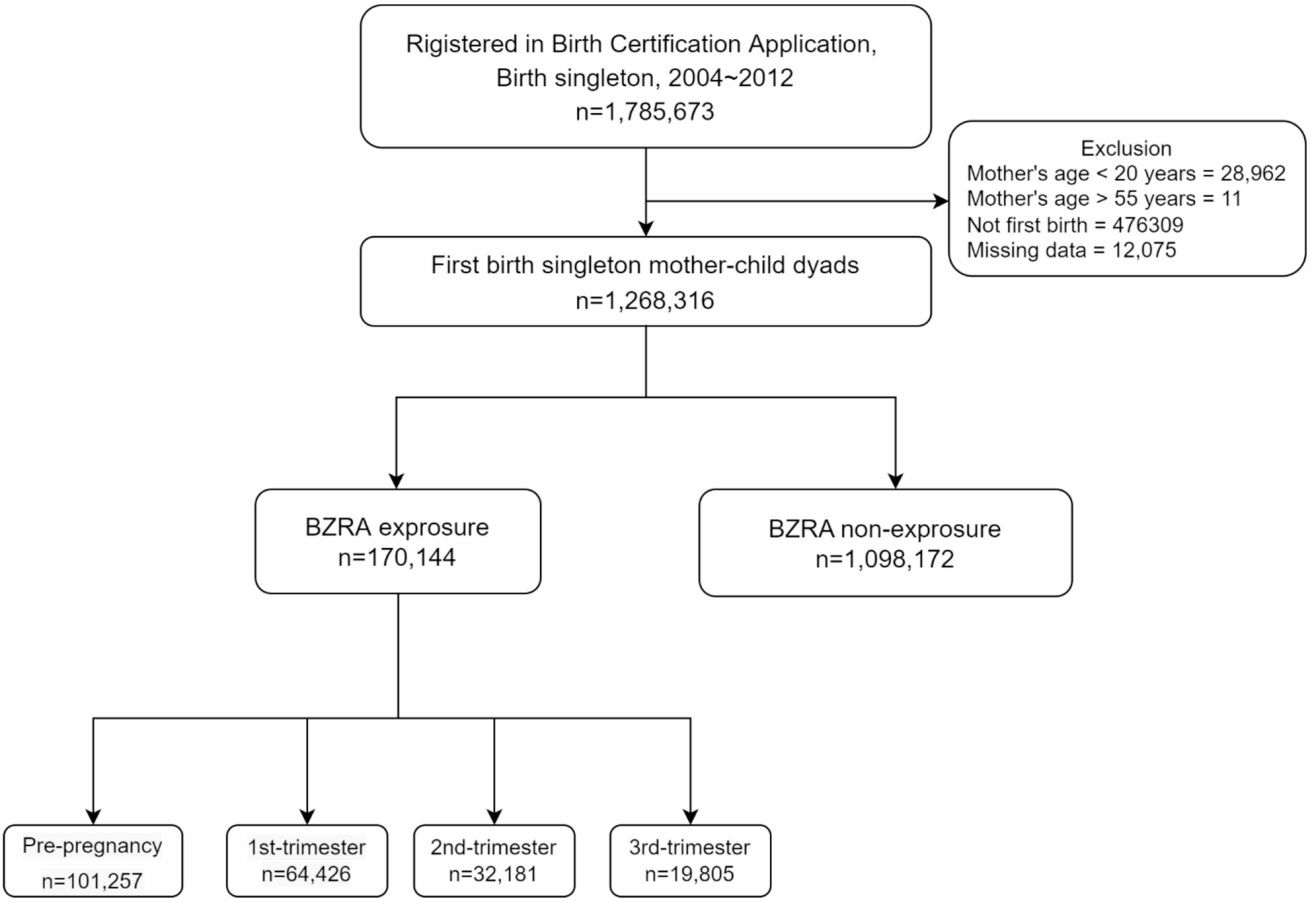
Patients’ inclusion steps

All pregnant women registered in the Taiwan Birth Certificate Application between January 1, 2004, and December 31, 2012, were included in this study. The database includes only live births and stillbirths at ≥ 20 weeks’ gestation or with a birth weight ≥ 500 g. To ensure independent observations, only the first delivery for each woman during the study period was included in the analysis. Multiple births and maternal age of < 20 or >55 years were excluded from the analysis because multiple births are considered at risk for adverse pregnancy outcomes, and fertility may end before menopause [23–26]. Gestational age was obtained from delivery records, and the conception date was estimated by subtracting gestational age from the delivery date. The pregnancy period was further classified into four intervals—preconception (90 days before conception), first trimester (week 0 to week 12), second trimester (week 13 to week 28), and third trimester (week 29 to delivery date). Maternal exposure to BZRAs was defined as having at least one prescription for a BZRA during the observation window. BZRA prescriptions were identified using Anatomical Therapeutic Chemical Classification System codes N05BA, N05CD, N03AE01, N05CF, A02AG, and A03CA. Prescription dates were used to assign each BZRA exposure to the appropriate window according to gestational age. Women with no BZRA prescriptions from 90 days before conception through delivery were classified as unexposed. Fig. 1 shows the flow diagram for the selection of study subjects.

### Outcome measures and confounders

The primary objective was to evaluate the association between BZRA exposure during each pregnancy trimester and adverse pregnancy outcomes. Pregnancy outcomes were obtained from Birth Certificate Application, including stillbirths (death of a fetus at > 20 weeks of gestation or weight of > 500 g), preterm births (gestational age of < 37 weeks), LBW (weight of < 2500 g), SGA (birth weight < 10th percentile for gestational age), cesarean section, 5-min Apgar score, and congenital malformations. The congenital malformations are recorded at delivery or within seven days postpartum and are classified into nine categories: (1) nervous system; (2) eye, ear, and face; (3) circulatory system; (4) digestive system; (5) urinary and genital system; (6) musculoskeletal system; (7) respiratory system; (8) chromosomal abnormalities; and (9) other congenital malformations (detailed definitions for congenital anomalies are provided in Supplementary Table 1).

Confounders were selected according to their clinical relevance to benzodiazepine indications and their potential impact on maternal and infant outcomes. Maternal variables included age, alcohol or nicotine dependence, psychiatric conditions (anxiety, insomnia, depression, schizophrenia, and bipolar disorder), and comorbidities such as hypertension, diabetes, gestational diabetes, hyperlipidemia, and epilepsy/seizures (Supplementary Table 1). Infant characteristics considered were birth date and sex.

### Statistical analysis

Demographic and clinical characteristics were summarized as mean (standard deviation) for continuous variables and number (percentage) for categorical variables. The balance of covariates between BZRA users and nonusers was assessed using standardized differences (SDiff), with an absolute SDiff ≥ 0.1 indicating meaningful imbalance [27]. Logistic regression models were used to calculate the crude odds ratios (ORs) and adjusted odds ratios (aORs) with 95% confidence intervals (95% CIs) of adverse pregnancy outcomes for BZRA users compared with those for nonusers. Moreover, stabilized inverse probability of treatment weighting (IPTW) was used to well-balance the demographic characteristics and comorbidities between the two groups [28]. Subgroup analyses were conducted according to common indications for BZRA use (anxiety, depression, and insomnia). All analyses were conducted using the SAS 9.4 software (SAS Institute, Inc., Cary, NC, USA), with a two-sided P value of < 0.05 indicating statistical significance.

## Results

Among 1,268,316 eligible mothers, 170,144 (13.4%) received at least one BZRA prescription from preconception to delivery. Specifically, BZRA exposure occurred in 101,257 (7.98%) during the preconception period, 64,426 (5.08%) during the first trimester, 32,181 (2.54%) during the second trimester, and 19,805 (1.56%) during the third trimester. The distribution of maternal characteristics and comorbidities is presented for the IPTW-weighted cohort in Table 1 and for the full cohort in Supplementary Table 2. B BZRA users had higher prevalences of psychiatric conditions than nonusers, including anxiety (11.18% vs. 1.67%), insomnia (18.83% vs. 3.85%), depression (5.91% vs. 0.63%), and bipolar disorder (0.8% vs. 0.06%) (all SDiffs > 0.1; Supplementary Table 2). After implementing the IPTW strategy, all baseline characteristics were well balanced between BZRA users and nonusers (all SDiffs < 0.1), resulting in similar distributions of demographic characteristics and comorbidities (Table 1).

**Table 1:** Maternal and offspring characteristics of BZRA users and nonusers in IPTW cohort

Characteristics	IPTW cohort analysis		
	BZRA users	Nonusers	SDiff ^a^
Maternal characteristics			
Age (mean±SD)	29.7 ± 4.8	29.7 ± 4.7	< 0.01
Anxiety & phobic	3.11%	3.47%	−0.02
Depression	1.46%	1.78%	−0.03
Insomnia & sleep disorder	6.03%	6.35%	−0.01
Maternal comorbidities			
Hypertension	3.53%	3.53%	< 0.01
Hyperlipidemia	1.13%	1.14%	< 0.01
Diabetes mellitus (DM)	7.31%	7.26%	< 0.01
Gestational DM	5.72%	5.68%	< 0.01
Schizophrenia	0.11%	0.19%	−0.02
Bipolar disorder	0.17%	0.28%	−0.02
Seizure & epilepsy	0.29%	0.29%	< 0.01
Nicotine dependence	0.19%	0.20%	< 0.01
Alcohol dependence	0.10%	0.11%	< 0.01
Offspring characteristics			
Sex (male)	52.17%	52.09%	< 0.01
Birth week	38.3 ± 2.2	38.4 ± 2.1	−0.05
Birth weight (gram)	3071.6 ± 503.7	3082.6 ± 491.0	−0.02
Childbirth year			0.04
2004	17.69%	15.75%	
2005	16.54%	14.03%	
2006	12.76%	12.46%	
2007	10.25%	11.18%	
2008	9.26%	10.04%	
2009	8.09%	9.41%	
2010	7.10%	7.82%	
2011	8.56%	8.87%	
2012	9.74%	10.44%	

*IPTW* inverse probability of treatment weights, *SDiff* standardized difference, *SD* standard deviation

^a^ SDiff of greater than 0.1 denotes meaningful imbalance in the baseline covariate

**Fig. 2:**
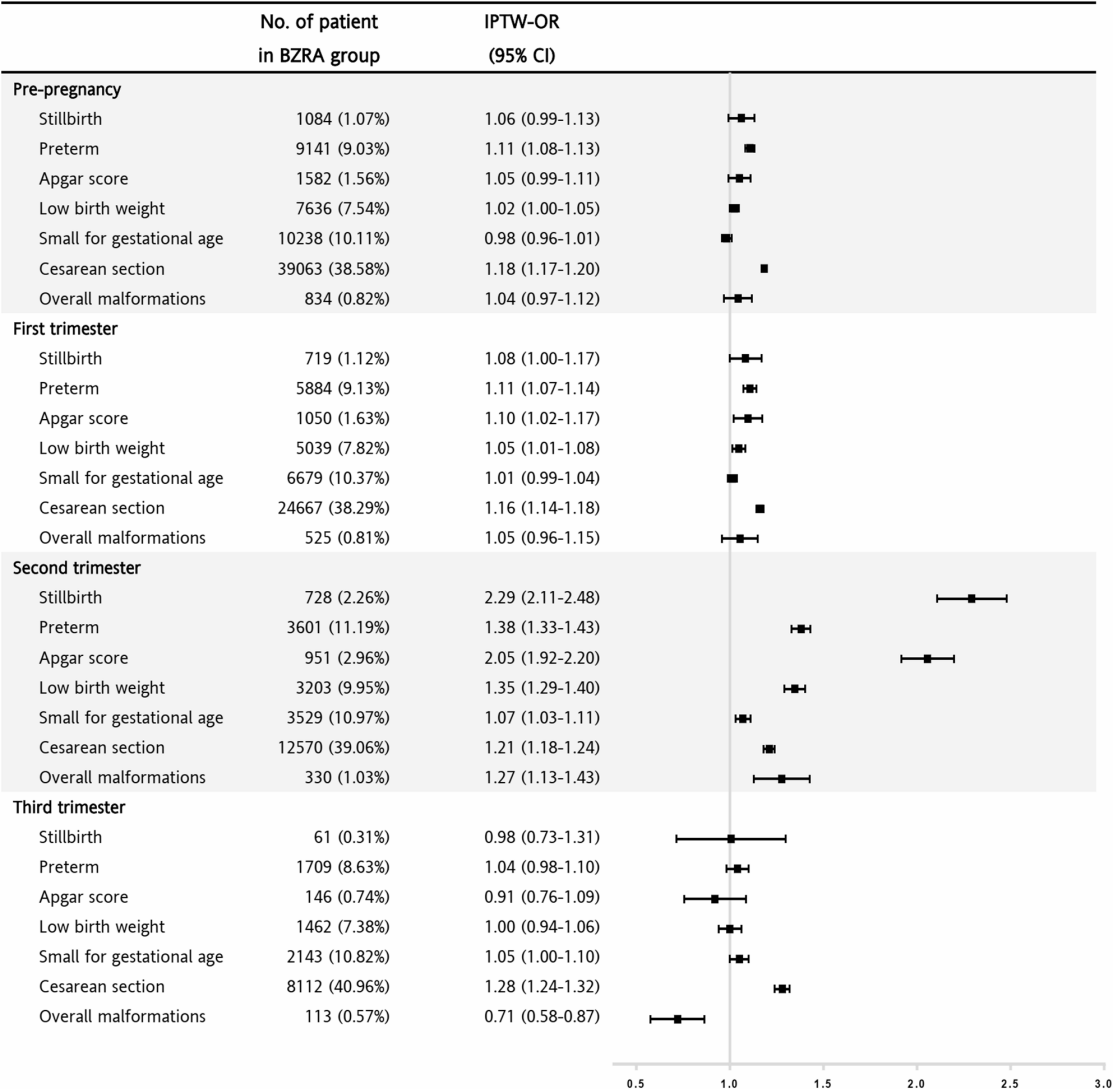
Risk of adverse pregnancy outcomes associated with maternal BZRA exposure in different trimesters (IPTW analysis) Note: All comparator estimates are based on the non-user group. Adjustment for mother’s age, child’s birth year, child’s sex, and mother’s comorbidities (hypertension, hyperlipidemia, diabetes mellitus and gestational diabetes mellitus); IPTW, inverse probability of treatment weights; BZRA, benzodiazepine receptor agonist; OR, odds ratio; CI, confidence interval

The risk of adverse pregnancy outcomes associated with maternal exposure to BZRAs in different trimesters is presented in Fig. 2 and Supplementary 3. During the pre-pregnancy period, the IPTW-ORs indicated that BZRA users had approximately 1.11- and 1.18-fold higher risks of preterm birth and cesarean section, respectively, compared with nonusers (all *P* < 0.001). In the first trimester, BZRA users had a significantly 1.11-, 1.10-, 1.05-, and 1.16-fold higher risk of preterm birth, Apgar score < 7, LBW, and cesarean section, respectively, than nonusers. Notably, exposure to BZRAs in the second trimester contributed to a significantly increased risk of all adverse pregnancy outcomes. Results from both multivariable-adjusted and IPTW models consistently showed significantly elevated risks of stillbirth (IPTW-OR: 2.29, 95% CI 2.11–2.48), preterm birth (IPTW-OR: 1.38, 95% CI 1.33–1.43), Apgar score < 7 (IPTW-OR: 2.05, 95% CI 1.92–2.20), LBW (IPTW-OR: 1.35, 95% CI 1.29–1.40), SGA (IPTW-OR: 1.07, 95% CI 1.03–1.11), cesarean section (IPTW-OR: 1.21, 95% CI 1.18–1.24), and overall congenital malformations (IPTW-OR: 1.27, 95% CI 1.13–1.43) after considering confounders. In the third trimester, exposure to BZRAs increased the risk of cesarean section (IPTW-OR: 1.28, 95% CI 1.24–1.32) but was associated with a lower risk of overall congenital malformations (IPTW-OR: 0.71, 95% CI 0.58–0.87). In addition, second-trimester BZRA exposure was significantly associated with higher odds of chromosomal abnormalities (IPTW-OR: 1.70, 95% CI 1.29–2.23) and congenital malformations in the nervous system (IPTW-OR: 1.59, 95% CI 1.10–2.30), digestive system (IPTW-OR: 1.51, 95% CI 1.05–2.19), and respiratory system (IPTW-OR: 2.53, 95% CI 1.45–4.39), as shown in Supplementary Fig. 1.

Then, Table 2 and Supplementary 4 presents the overall associations between maternal BZRA exposure and adverse pregnancy outcomes. The IPTW-ORs for BZRA users consistently indicated that maternal exposure to BZRAs was associated with a high risk of stillbirth (IPTW-OR: 1.19, 95% CI 1.14–1.25), preterm birth (IPTW-OR:1.11, 95% CI 1.09–1.13), Apgar score < 7 (IPTW-OR:1.17, 95% CI 1.12–1.22), LBW (IPTW-OR:1.05, 95% CI 1.03–1.07), and cesarean section (IPTW-OR:1.15, 95% CI 1.14–1.17). Subgroup analyses were conducted to further stratify the study sample by common indications for BZRA use, including anxiety, depression, and insomnia (Supplementary Table 5). The increased risks of adverse pregnancy outcomes associated with BZRA exposure were mainly observed among women without these psychiatric comorbidities. In these subgroups, BZRA exposure remained consistently associated with higher odds of stillbirth, preterm birth, low Apgar score, LBW, and cesarean section after IPTW adjustment. In contrast, among women with anxiety, depression, or insomnia, most associations were not statistically significant. This pattern suggests that the apparent risk elevation may be more pronounced in women without underlying mental health disorders, whereas the associations appeared attenuated among those with these comorbidities, possibly reflecting differences in baseline risk profiles.

**Table 2:** Risk of adverse pregnancy outcomes with maternal BZRA use in IPTW cohort

Pregnancy outcomes	BZRA group (*n*, %)	Nonusers (*n*, %)	IPTW-OR (95% CI) ^a^
Stillbirth	1992 (1.17%)	10,351 (0.94%)	1.19 *** (1.14–1.25)
Preterm	15 193 (8.93%)	82,652 (7.53%)	1.11 *** (1.09–1.13)
Apgar score < 7 (5 min)	2867 (1.69%)	15,152 (1.38%)	1.17 *** (1.12–1.22)
Low birth weight	12 955 (7.61%)	74,587 (6.79%)	1.05 *** (1.03–1.07)
Small for gestational age	17 369 (10.21%)	108,403 (9.87%)	1.00 (0.99–1.02)
Cesarean section	64 003 (37.62%)	365,030 (33.24%)	1.15 *** (1.14–1.17)
All congenital malformations	1416 (0.83%)	8289 (0.75%)	1.06 (1.00–1.12.00.12)
Nervous system	121 (0.07%)	657 (0.06%)	1.18 (0.97–1.43)
Eye-ear-face	293 (0.17%)	1774 (0.16%)	1.04 (0.91–1.17)
Circulatory	187 (0.11%)	1092 (0.1%)	1.06 (0.90–1.24)
Digestive	129 (0.08%)	693 (0.06%)	1.13 (0.93–1.38)
Urinary and genital	134 (0.08%)	829 (0.08%)	1.03 (0.86–1.24)
Musculoskeletal	322 (0.19%)	1909 (0.17%)	1.05 (0.93–1.18)
Respiratory	41 (0.02%)	190 (0.02%)	1.20 (0.84–1.72)
Chromosomal abnormalities	185 (0.11%)	1168 (0.11%)	0.99 (0.85–1.16)
Others	122 (0.07%)	745 (0.07%)	0.94 (0.77–1.15)

*BZRA* benzodiazepine receptor agonist, *IPTW* inverse probability of treatment weights, *OR* odds ratio, *CI* confidence interval

^a^ Considering for mother’s age, child’s birth year, child’s sex, and mother’s comorbidities (hypertension, hyperlipidemia, diabetes mellitus and gestational diabetes mellitus)

* *P* < 0.05

*** *P* < 0.001

## Discussion

Our study found that maternal BZRA use was associated with increased risks of stillbirth, preterm birth, low Apgar score, LBW, and cesarean delivery. By exposure period, pre-pregnancy and first-trimester use were associated with higher risks of preterm birth, LBW, and cesarean delivery. Second-trimester exposure showed the most pronounced associations, including elevated risks of stillbirth, preterm birth, low Apgar score, LBW, SGA, cesarean delivery, and overall congenital malformations as well as specific nervous, digestive, respiratory anomalies and chromosomal abnormalities. Third-trimester exposure was mainly associated with an increased risk of cesarean delivery. To the best of our knowledge, this is the first population-based study to comprehensively evaluate BZRA exposure across different pregnancy periods in relation to adverse obstetric and neonatal outcomes. We observed a relatively low prevalence of congenital anomalies (0.83% among the exposure group and 0.75% among the unexposed group), which was consistent with findings from previous Taiwanese studies [29].

Although only a limited number of studies have examined trimester-specific BZRA exposure, our findings are generally consistent with the available evidence. Prior research has linked maternal BZRA use to an increased risk of preterm birth, LBW and SGA, with some studies reporting higher cesarean delivery rates among exposed women [22, 30, 31]. However, most previous investigations have focused on exposure during the entire pregnancy or only the first trimester. With regard to congenital malformations, our results align with prior meta-analyses indicating no clear increase in overall risk with first-trimester BZRA exposure. A few studies have also noted an association with digestive system defects [30, 32], but two meta-analyses of cohort studies found no significant association between first-trimester BZRA exposure and congenital malformations [22, 33]. Pooled analyses of cohort and case–control studies also showed similar null findings [34, 35]. Overall, BZRA use does not appear to have strong teratogenic potential, but the observed associations with other adverse pregnancy outcomes warrant clinical attention.

Several pharmacological mechanisms could help explain these associations. BZRAs enhance GABA_A_-mediated inhibitory signaling, which may modulate maternal vasopressin and oxytocin release [36, 37]. Such neuroendocrine effects could alter uterine contractility and placental perfusion, potentially contributing to impaired fetal growth and the observed associations with SGA and LBW [38, 39]. Reduced uteroplacental perfusion may also lead to chronic fetal hypoxia, thereby increasing the risk of preterm delivery or stillbirth [39–41]. BZRAs also cross the placenta and can accumulate in the fetus, where their half-life is substantially longer than in the mother, leading to higher fetal drug concentrations [42]. The second trimester (weeks 13–28) is a critical stage characterized by active neuronal proliferation and differentiation, with GABAergic signaling playing an important regulatory role [20, 43]. Animal studies show that benzodiazepines can disrupt this signaling and trigger apoptotic neurodegeneration during the brain growth-spurt period [44]. These mechanisms provide a biologically plausible explanation for the heightened risks we observed across trimesters, underscoring the need for caution when prescribing BZRAs throughout gestation.

This study has many strengths. First, this is the first investigation to comprehensively examine the use of BZRAs in different pregnancy trimesters and the risk of adverse pregnancy outcomes. Second, the sample size was much larger than that in previous studies, helping us detect rare and specific congenital malformation outcomes. Third, the main findings were consistent in the two analytic strategies, multivariable adjustment and IPTW weighting, which helped reduce potential confounding and bias. However, there were several limitations that should be considered when interpreting the study findings. First, the NHI database captures dispensing records but does not contain data on medication consumption or patient adherence. Therefore, adherence to benzodiazepine therapy could not be assessed. Second, although we adjusted for maternal comorbidities and applied IPTW methods, residual confounding cannot be ruled out. Important variables such as family history, laboratory data (e.g., folic acid levels and blood pressure), maternal body mass index or obesity status, lifestyle factors (e.g., sleep quality, stress, and exercise habits), socioeconomic status (e.g., income and education), and exposure to other teratogenic drugs were not incorporated in this study. Third, because the study population was based entirely in Taiwan, the findings may not be fully generalizable to populations in other regions with different genetic backgrounds and healthcare systems. Fourth, dose–response analyses were not conducted, limiting the assessment of potential exposure–response relationships. Fifth, pregnancies that ended before 20 weeks of gestation, including miscarriages and medical terminations, were not analysed in this study; therefore, outcomes related to early pregnancy loss and congenital anomalies that might have resulted in such losses could not be assessed, and their prevalence may be underestimated. In addition, time-to-event models such as Poisson or survival analyses were not employed in this study to account for differences in pregnancy duration. Therefore, differences in follow-up time between stillbirths and full-term pregnancies may have led to differential exposure assessment. Finally, the study data spanned 2004–2012, and clinical practice and prescribing patterns regarding BZRAs may have changed over time; therefore, the findings may not fully represent current clinical practice. Future studies are warranted to address these limitations and further clarify the associations observed.

## Conclusions

In this nationwide study, we observed a small but statistical association between BZRA exposure and adverse pregnancy outcomes, which extended from the pre-pregnancy period through all trimesters. Given the study’s observational design, causality cannot be inferred, and the potential for residual confounding warrants a cautious interpretation of these results. This finding does not suggest avoiding necessary treatment, but rather highlights the need for a thorough discussion between clinicians and patients, carefully weighing the risks and benefits of BZRA therapy during family planning and pregnancy.

## Supplementary Information

Supplementary Material 1.

Supplementary Material 2.

Supplementary Material 3.

Supplementary Material 4.

Supplementary Material 5.

Supplementary Material 6.

## Data Availability

Data used in this study are handled and stored by the Health and Welfare Data Science Center. Interested researchers can obtain the data through formal application to the Health and Welfare Data Science Center, Department of Statistics, Ministry of Health and Welfare, Taiwan (https://www.mohw.gov.tw/mp-2.html).

## Abbreviations

BZRA: Benzodiazepine receptor agonist
IPTW: Inverse probability of treatment weighting
OR: Odds ratio
LBW: Low birth weights
GABA: Gamma-aminobutyric acid
SGA: Small for gestational age
SDiff: Standardized difference
CI: Confidence intervals

## References

[1] Boekhorst MG, Beerthuizen A, Endendijk JJ, Van Broekhoven KE, Van Baar A, Bergink V, Pop VJ. Different trajectories of depressive symptoms during pregnancy. J Affect Disord. 2019;248:139–46. 30731281 10.1016/j.jad.2019.01.021

[2] Kjeldgaard HK, Vikanes Å, Benth JŠ, Junge C, Garthus-Niegel S, Eberhard-Gran M. The association between the degree of nausea in pregnancy and subsequent posttraumatic stress. Arch Womens Ment Health. 2019;22(4):493–501. 30225528 10.1007/s00737-018-0909-z PMC6647437

[3] Urizar GG, Muñoz RF. Role of maternal depression on child development: a prospective analysis from pregnancy to early childhood. Child Psychiatry Hum Dev. 2022;53(3):502–14. 33646485 10.1007/s10578-021-01138-1 PMC10911822

[4] Tuovinen S, Lahti-Pulkkinen M, Girchenko P, Lipsanen J, Lahti J, Heinonen K, Reynolds RM, Hämäläinen E, Kajantie E, Laivuori H. Maternal depressive symptoms during and after pregnancy and child developmental milestones. Depress Anxiety. 2018;35(8):732–41. 29667739 10.1002/da.22756

[5] Insan N, Weke A, Forrest S, Rankin J. Social determinants of antenatal depression and anxiety among women in South asia: a systematic review & meta-analysis. PLoS One. 2022;17(2):e0263760. 35139136 10.1371/journal.pone.0263760 PMC8827460

[6] Raffi ER, Nonacs R, Cohen LS. Safety of psychotropic medications during pregnancy. Clin Perinatol. 2019;46(2):215–34. 31010557 10.1016/j.clp.2019.02.004

[7] Calderon-Margalit R, Qiu C, Ornoy A, Siscovick DS, Williams MA. Risk of preterm delivery and other adverse perinatal outcomes in relation to maternal use of psychotropic medications during pregnancy. Am J Obstet Gynecol. 2009;201(6):579. e571-579. e578. 10.1016/j.ajog.2009.06.061 PMC2881461 19691950

[8] Richards J, Möhler H. Benzodiazepine receptors. Neuropharmacology. 1984;23(2):233–42. 6324017 10.1016/0028-3908(84)90064-9

[9] Brandt J, Bressi J, Lê ML, Neal D, Cadogan C, Witt-Doerring J, Witt-Doerring M, Wright S. Prescribing and deprescribing guidance for benzodiazepine and benzodiazepine receptor agonist use in adults with depression, anxiety, and insomnia: an international scoping review. EClinicalMedicine. 2024;70:102507. 38516102 10.1016/j.eclinm.2024.102507 PMC10955669

[10] Wick J. The history of benzodiazepines. Consultant Pharmacist^®^. 2013;28(9):538–48. 10.4140/TCP.n.2013.538 24007886

[11] Bais B, Molenaar NM, Bijma HH, Hoogendijk WJ, Mulder CL, Luik AI, et al. Prevalence of benzodiazepines and benzodiazepine-related drugs exposure before, during and after pregnancy: a systematic review and meta-analysis. J Affect Disord. 2020;269:18–27. 32217339 10.1016/j.jad.2020.03.014

[12] Lin YH, Chen MH, Chang YC, Chen L, Hsiung CA, Wu SI: Prevalence of exposure to benzodiazepines among pregnant women in Taiwan: A nationwide longitudinal study. J Sleep Res. 2022;31(6):e13678. 10.1111/jsr.13678 PMC9788177 35775446

[13] Juric S, Newport DJ, Ritchie JC, Galanti M, Stowe ZN. Zolpidem (Ambien^®^) in pregnancy: placental passage and outcome. Arch Women Ment Health. 2009;12(6):441–6. 10.1007/s00737-009-0100-7 19657707

[14] Saito J, Ishii M, Mito A, Yakuwa N, Kawasaki H, Tachibana Y, et al. Etizolam levels in maternal serum, cord blood, and breast milk during pregnancy and lactation: a case report. Psychiatry Clin Neurosci. 2021;75(6):211–2. 33733552 10.1111/pcn.13216

[15] Saito J, Ishii M, Miura Y, Yakuwa N, Kawasaki H, Suzuki T, et al. Brotizolam during pregnancy and lactation: brotizolam levels in maternal serum, cord blood, breast milk, and neonatal serum. Breastfeed Med. 2021;16(7):579–82. 33666494 10.1089/bfm.2021.0013

[16] Wang X, Zhang T, Ekheden I, Chang Z, Hellner C, Hasselström J, Jayaram-Lindström N, D’Onofrio BM, Larsson H, Mataix-Cols D. Prenatal exposure to benzodiazepines and Z-drugs in humans and risk of adverse neurodevelopmental outcomes in offspring: A systematic review. Neurosci Biobehavioral Reviews. 2022;137: 104647. 10.1016/j.neubiorev.2022.104647 35367514

[17] Bleyl SB, Schoenwolf GC: What is the timeline of important events during pregnancy that may be disrupted by a teratogenic exposure? Teratology Primer. The Teratology Society; 2010. Available from: https://birthdefectsresearch.org/primer/PrimerPDF/What-Is-the-Timeline-of-Important-Events-During-Pregnancy-That-May-Be-Disrupted-by-a-Teratogenic-Exposure.pdf

[18] Noh Y, Lee H, Choi A, Kwon JS, Choe S-A, Chae J, et al. First-trimester exposure to benzodiazepines and risk of congenital malformations in offspring: a population-based cohort study in South Korea. PLoS Med. 2022;19(3):e1003945. 35235572 10.1371/journal.pmed.1003945 PMC8926183

[19] Tinker SC, Reefhuis J, Bitsko RH, Gilboa SM, Mitchell AA, Tran EL, Werler MM, Broussard CS, Study NBDP. Use of benzodiazepine medications during pregnancy and potential risk for birth defects, National birth defects prevention Study, 1997–2011. Birth Defects Res. 2019;111(10):613–20. 30891943 10.1002/bdr2.1497 PMC7186570

[20] Schoenwolf GC, Bleyl SB, Brauer PR, Francis-West PH: Larsen’s human embryology. 5th ed. Philadelphia (PA): Elsevier/Churchill Livingstone (Elsevier Health Sciences); 2014.

[21] Mullis PE, Tonella P. Regulation of fetal growth: consequences and impact of being born small. Best Pract Res Clin Endocrinol Metab. 2008;22(1):173–90. 18279787 10.1016/j.beem.2007.07.010

[22] Grigoriadis S, Alibrahim A, Mansfield JK, Sullovey A, Robinson GE. Hypnotic benzodiazepine receptor agonist exposure during pregnancy and the risk of congenital malformations and other adverse pregnancy outcomes: A systematic review and meta-analysis. Acta Psychiatrica Scandinavica. 2022;146(4):312–24. 10.1111/acps.13441 35488412

[23] Bateman BT, Simpson LL. Higher rate of stillbirth at the extremes of reproductive age: a large nationwide sample of deliveries in the United States. Am J Obstet Gynecol. 2006;194(3):840–5. 16522422 10.1016/j.ajog.2005.08.038

[24] Alexander GR, Salihu HM: Perinatal outcomes of singleton and multiple births in the United States, 1995–98. In: Blickstein I, Keith LG, editors. Prenatal Assessment of Multiple Pregnancy. CRC Press; 2018. p. 33–40.

[25] Yogev Y, Melamed N, Bardin R, Tenenbaum-Gavish K, Ben-Shitrit G, Ben-Haroush A. Pregnancy outcome at extremely advanced maternal age. Am J Obstet Gynecol. 2010;203(6):558. e551-558. e557. 10.1016/j.ajog.2010.07.039 20965486

[26] Broekmans FJ, Faddy MJ, Scheffer G, te Velde ER. Antral follicle counts are related to age at natural fertility loss and age at menopause. Menopause. 2004;11(6 Part 1 of 2):607–14. 15545788 10.1097/01.gme.0000123643.76105.27

[27] Austin PC. Balance diagnostics for comparing the distribution of baseline covariates between treatment groups in propensity-score matched samples. Stat Med. 2009;28(25):3083–107. 19757444 10.1002/sim.3697 PMC3472075

[28] Desai RJ, Franklin JM. Alternative approaches for confounding adjustment in observational studies using weighting based on the propensity score: a primer for practitioners. *bmj* . 2019;367:l5657. 10.1136/bmj.l5657 31645336

[29] Lin KM, Yang YH, Lee CP, Chen KJ, Yang YH, Sheen JM, Chien SJ. Maternal and neonatal outcomes in women with congenital heart disease: A nationwide population-based study. J Formos Med Assoc. 2024;123(7):744–50. 38485554 10.1016/j.jfma.2023.11.009

[30] Wikner BN, Stiller CO, Bergman U, Asker C, Källén B. Use of benzodiazepines and benzodiazepine receptor agonists during pregnancy: neonatal outcome and congenital malformations. Pharmacoepidemiol Drug Saf. 2007;16(11):1203–10. 17894421 10.1002/pds.1457

[31] Wang LH, Lin HC, Lin CC, Chen YH, Lin HC. Increased risk of adverse pregnancy outcomes in women receiving Zolpidem during pregnancy. Clin Pharmacol Ther. 2010;88(3):369–74. 20686480 10.1038/clpt.2010.97

[32] Bonnot O, Vollset S, Godet P, d’Amato T, Dalery J, Robert E. In utero exposure to benzodiazepine. Is there a risk for anal Atresia with lorazepam? L’encephale. 2003;29(6):553–9. 15029090

[33] Grigoriadis S, Graves L, Peer M, Mamisashvili L, Dennis C-L, Vigod SN, Steiner M, Brown C, Cheung A, Dawson H. Benzodiazepine use during pregnancy alone or in combination with an antidepressant and congenital malformations: systematic review and meta-analysis. J Clin Psychiatry. 2019;80(4):1845. 10.4088/JCP.18r12412 31294935

[34] Enato E, Moretti M, Koren G. Motherisk rounds: the fetal safety of benzodiazepines: an updated meta-analysis. J Obstet Gynaecol Can. 2011;33(1):46–8. 21272436 10.1016/S1701-2163(16)34772-7

[35] Dolovich LR, Addis A, Vaillancourt JR, Power JB, Koren G, Einarson TR. Benzodiazepine use in pregnancy and major malformations or oral cleft: meta-analysis of cohort and case-control studies. BMJ. 1998;317(7162):839–43. 9748174 10.1136/bmj.317.7162.839 PMC31092

[36] Catelli JM, Giakas WJ, Sved AF. GABAergic mechanisms in nucleus tractus solitarius alter blood pressure and vasopressin release. Brain Res. 1987;403(2):279–89. 3828822 10.1016/0006-8993(87)90065-5

[37] Yagi K, Onaka T. A benzodiazepine, chlordiazepoxide, blocks vasopressin and Oxytocin release after footshocks but not osmotic stimulus in the rat. Neurosci Lett. 1996;203(1):49–52. 8742044 10.1016/0304-3940(95)12262-1

[38] Arenas GA, Lorca RA. Effects of hypoxia on uteroplacental and fetoplacental vascular function during pregnancy. Front Physiol. 2024;15:1490154. 39744703 10.3389/fphys.2024.1490154 PMC11688409

[39] Ghi T, Fieni S, Ramirez Zegarra R, Pereira S, Dall’Asta A, Chandraharan E. Relative uteroplacental insufficiency of labor. Acta Obstet Gynecol Scand. 2024;103(10):1910–8. 39107951 10.1111/aogs.14937 PMC11426226

[40] Sedeek M, Gilbert JS, LaMarca BB, Sholook M, Chandler DL, Wang Y, Granger JP. Role of reactive oxygen species in hypertension produced by reduced uterine perfusion in pregnant rats. Am J Hypertens. 2008;21(10):1152–6. 18670418 10.1038/ajh.2008.239 PMC2786058

[41] Woods JR, Plessinger MA, Clark KE. Effect of cocaine on uterine blood flow and fetal oxygenation. JAMA. 1987;257(7):957–61. 3806879

[42] Gamble J, Moore J, Lamki H, Howard P. A study of plasma diazepam levels in mother and infant. BJOG Int J Obstet Gynaecol. 1977;84(8):588–91. 10.1111/j.1471-0528.1977.tb12659.x 889746

[43] Leibovitz Z, Lerman-Sagie T, Haddad L. Fetal brain development: regulating processes and related malformations. Life (Basel). 2022;12(6):809. 35743840 10.3390/life12060809 PMC9224903

[44] Olney JW, Wozniak DF, Jevtovic-Todorovic V, Farber NB, Bittigau P, Ikonomidou C. Drug-induced apoptotic neurodegeneration in the developing brain. Brain Pathol. 2002;12(4):488–98. 12408236 10.1111/j.1750-3639.2002.tb00467.x PMC8095833

