# Supplementary material for: Use of benzodiazepine receptor agonists in different pregnancy trimesters and risk of maternal and neonatal outcomes: a propensity weighted cohort study in Taiwan
Source: BMC Pregnancy Childbirth. 2025 Dec 6;25:1344. doi: 10.1186/s12884-025-08549-1 (PMC12751940; doi:10.1186/s12884-025-08549-1)
Supplement: Supplementary file 1 — Supplementary Material 1. [file 12884_2025_8549_MOESM1_ESM.docx]

**Supplementary Table 1. Definitions of congenital malformations and maternal covariates.**

| **Variable** | **Definition** | **Assessment period** |
| --- | --- | --- |
| **Stillbirth** | Coding in Birth Certificate Application | At delivery |
| **Preterm** | Coding in Birth Certificate Application | At delivery |
| **Apgar score < 7 (5min)** | Coding in Birth Certificate Application | At delivery |
| **Low birth weight** | Coding in Birth Certificate Application | At delivery |
| **Small for gestational age** | Coding in Birth Certificate Application | At delivery |
| **Congenital malformations** | Coding in Birth Certificate Application |  |
| **Nervous system** | CGD_0100–0109 | Within 7 days after delivery |
| **Eye, ear, and face** | CGD_0200–0208 | Within 7 days after delivery |
| **Circulatory system** | CGD_0300–0310 | Within 7 days after delivery |
| **Digestive system** | CGD_0400–0412 | Within 7 days after delivery |
| **Urinary and genital system** | CGD_0500–0507 | Within 7 days after delivery |
| **Musculoskeletal system** | CGD_0600–0608 | Within 7 days after delivery |
| **Respiratory system** | CGD_0700–0713 | Within 7 days after delivery |
| **Chromosomal abnormalities** | CGD_0800–0807 | Within 7 days after delivery |
| **Other congenital malformations** | CGD_0900–0907 | Within 7 days after delivery |
| **Preterm birth** | Gestational age <37 weeks (from birth reporting system) | At delivery |
| **Low birth weight** | Birth weight <2,500 g (from birth reporting system) | At delivery |
| **5-min Apgar score** | <7 classified as low (from birth reporting system) | At delivery |

**Supplementary Table 1. Definitions of congenital malformations and maternal covariates. (Continued)**

| **Variable** | **Definition** | **Assessment period** |
| --- | --- | --- |
| **Maternal covariates** |  |  |
| **Anxiety disorder** | 293.84, 300.00, 300.01, 300.02, 300.2, 300.09, 313.0 | 1 year before conception to delivery |
| **Insomnia** | 780.50, 780.51, 780.52, 307.40, 307.41, 307.42 | 1 year before conception to delivery |
| **Hypertension** | 401–405, 642 | 1 year before conception to delivery |
| **Diabetes** | 250, 6480 | 1 year before conception to delivery |
| **Hyperlipidemia** | 272 | 1 year before conception to delivery |
| **Depression** | 301.12, 300.4, 309.0, 309.1, 311, 296.2, 296.3 | 1 year before conception to delivery |
| **Schizophrenia** | 295 | 1 year before conception to delivery |
| **Bipolar disorder** | 296.1, 296.4–296.8 (except 296.82) | 1 year before conception to delivery |
| **Epilepsy** | 345, 7803 | 1 year before conception to delivery |
| **Gestational diabetes** | 6488 | From 20 weeks of gestation to delivery |
| **Smoking** | V15.82, 989.84, 305.1 | 1 year before conception to delivery |
| **Alcohol dependence** | 303, 291 | 1 year before conception to delivery |
