# Supplementary material for: Use of benzodiazepine receptor agonists in different pregnancy trimesters and risk of maternal and neonatal outcomes: a propensity weighted cohort study in Taiwan
Source: BMC Pregnancy Childbirth. 2025 Dec 6;25:1344. doi: 10.1186/s12884-025-08549-1 (PMC12751940; doi:10.1186/s12884-025-08549-1)
Supplement: Supplementary file 2 — Supplementary Material 2. [file 12884_2025_8549_MOESM2_ESM.docx]

**Supplementary table 2. Maternal and offspring characteristics of BZRA users and nonusers in Full cohort.**

| **Characteristics** | **Full cohort analysis** | | |
| --- | --- | --- | --- |
|  | **BZRA users (***n*=170 144**)** | **Nonusers** (*n*=1 098 172) | **SDiff** ^a^ |
| Maternal characteristics |  |  |  |
| Age | 29.6 ± 4.8 | 29.7 ± 4.7 | -0.04 |
| Anxiety & phobic | 19 019 (11.18%) | 18 318 (1.67%) | 0.40 |
| Depression | 10 054 (5.91%) | 6942 (0.63%) | 0.30 |
| Insomnia & sleep disorder | 32 042 (18.83%) | 42 306 (3.85%) | 0.49 |
| Maternal comorbidities |  |  |  |
| Hypertension | 7917 (4.65%) | 36 421 (3.32%) | 0.07 |
| Hyperlipidemia | 3139 (1.84%) | 10 960 (1.00%) | 0.07 |
| Diabetes mellitus (DM) | 12 716 (7.47%) | 79 352 (7.23%) | 0.01 |
| Gestational DM | 9091 (5.34%) | 62 962 (5.73%) | -0.02 |
| Schizophrenia | 864 (0.51%) | 432 (0.04%) | 0.09 |
| Bipolar disorder | 1361 (0.80%) | 621 (0.06%) | 0.11 |
| Seizure & epilepsy | 1130 (0.66%) | 2425 (0.22%) | 0.07 |
| Nicotine dependence | 779 (0.46%) | 1718 (0.16%) | 0.05 |
| Alcohol dependence | 584 (0.13%) | 518 (0.05%) | 0.07 |
| Offspring characteristics |  |  |  |
| Sex (male) | 88 890 (52.24%) | 571 806 (52.07%) | <0.01 |
| Birth week | 38.2 ± 2.3 | 38.26 ± 2.11 | -0.07 |
| Birth weight (gram) | 3061.7 ± 511.3 | 3084.2 ± 488.8 | -0.04 |
| Childbirth year |  |  |  |
| 2004 | 30100 (17.69) | 172919 (15.75) | 0.130 |
| 2005 | 28144 (16.54) | 154095 (14.03) |  |
| 2006 | 21717 (12.76) | 136852 (12.46) |  |
| 2007 | 17442 (10.25) | 122786 (11.18) |  |
| 2008 | 15751 (9.26) | 110258 (10.04) |  |
| 2009 | 13757 (8.09) | 103321 (9.41) |  |
| 2010 | 12085 (7.1) | 85905 (7.82) |  |
| 2011 | 14572 (8.56) | 97385 (8.87) |  |
| 2012 | 16576 (9.74) | 114651 (10.44) |  |

**Note:** ^a^ SDiff of greater than 0.1 denotes meaningful imbalance in the baseline covariate; SDiff, standardized difference.
