# Supplementary material for: Use of benzodiazepine receptor agonists in different pregnancy trimesters and risk of maternal and neonatal outcomes: a propensity weighted cohort study in Taiwan
Source: BMC Pregnancy Childbirth. 2025 Dec 6;25:1344. doi: 10.1186/s12884-025-08549-1 (PMC12751940; doi:10.1186/s12884-025-08549-1)
Supplement: Supplementary file 3 — Supplementary Material 3. [file 12884_2025_8549_MOESM3_ESM.docx]

|  | **Crude OR (95% CI)** | **Adjusted OR (95% CI) ^a^** |
| --- | --- | --- |
| **Pre-pregnancy** |  |  |
| Stillbirth | 1.14*** (1.07-1.21) | 1.08* (1.01-1.15) |
| Preterm | 1.22*** (1.19-1.25) | 1.12*** (1.09-1.14) |
| Apgar score | 1.13*** (1.08-1.2) | 1.06 (1-1.12) |
| Low birth weight | 1.12*** (1.09-1.15) | 1.04** (1.01-1.07) |
| Small for gestational age | 1.03* (1.01-1.05) | 1.00 (0.98-1.02) |
| Cesarean section | 1.26*** (1.25-1.28) | 1.17*** (1.15-1.19) |
| Overall malformations | 1.09* (1.02-1.17) | 1.06 (0.98-1.14) |
| **First trimester** |  |  |
| Stillbirth | 1.19*** (1.1-1.28) | 1.12** (1.03-1.22) |
| Preterm | 1.24*** (1.2-1.27) | 1.14*** (1.10-1.17) |
| Apgar score | 1.18*** (1.11-1.26) | 1.10** (1.03-1.18) |
| Low birth weight | 1.16*** (1.13-1.2) | 1.08*** (1.05-1.12) |
| Small for gestational age | 1.06*** (1.03-1.08) | 1.02 (0.99-1.05) |
| Cesarean section | 1.25*** (1.23-1.27) | 1.18*** (1.16-1.20) |
| Overall malformations | 1.08 (0.99-1.18) | 1.06 (0.96-1.16) |
| **Second trimester** |  |  |
| Stillbirth | 2.43*** (2.25-2.62) | 2.27*** (2.09-2.47) |
| Preterm | 1.55*** (1.49-1.6) | 1.42*** (1.37-1.47) |
| Apgar score | 2.18*** (2.04-2.33) | 2.01*** (1.87-2.16) |
| Low birth weight | 1.52*** (1.46-1.57) | 1.39*** (1.34-1.45) |
| Small for gestational age | 1.13*** (1.09-1.17) | 1.08*** (1.04-1.12) |
| Cesarean section | 1.29*** (1.26-1.32) | 1.24*** (1.21-1.26) |
| Overall malformations | 1.36*** (1.22-1.52) | 1.30*** (1.16-1.45) |
| **Third trimester** |  |  |
| Stillbirth | 1.21 (0.94-1.57) | 1.08 (0.82-1.40) |
| Preterm | 1.28*** (1.22-1.34) | 1.14*** (1.08-1.20) |
| Apgar score | 1.06 (0.9-1.25) | 0.92 (0.78-1.10) |
| Low birth weight | 1.22*** (1.15-1.29) | 1.08** (1.03-1.15) |
| Small for gestational age | 1.12*** (1.07-1.17) | 1.06* (1.02-1.11) |
| Cesarean section | 1.39*** (1.35-1.43) | 1.31*** (1.27-1.35) |
| Overall malformations | 0.76** (0.63-0.91) | 0.74** (0.61-0.89) |

**Supplementary table 3. Risk of adverse pregnancy outcomes associated with maternal BZRA exposure in different trimesters (Full cohort analysis)**

**Note:** ^a^ Considering for mother’s age, child’s birth year, child’s sex, and mother’s comorbidities (hypertension, hyperlipidemia, diabetes mellitus and gestational diabetes mellitus); BZRA, benzodiazepine receptor agonist; OR, odds ratio; CI, confidence interval;
* P < 0.05, ** P < 0.01, *** P < 0.001
