# Supplementary material for: Use of benzodiazepine receptor agonists in different pregnancy trimesters and risk of maternal and neonatal outcomes: a propensity weighted cohort study in Taiwan
Source: BMC Pregnancy Childbirth. 2025 Dec 6;25:1344. doi: 10.1186/s12884-025-08549-1 (PMC12751940; doi:10.1186/s12884-025-08549-1)
Supplement: Supplementary file 4 — Supplementary Material 4. [file 12884_2025_8549_MOESM4_ESM.docx]

**Supplementary Table 4. Risk of adverse pregnancy outcomes with maternal BZRA use in Full cohort.**

| **Pregnancy outcomes** | **Crude OR (95% CI)** | **Adjusted OR (95% CI) ^a^** |
| --- | --- | --- |
| Stillbirth | 1.25 *** (1.19-1.30) | 1.20 *** (1.14-1.27) |
| Preterm | 1.21 *** (1.18-1.23) | 1.13 *** (1.11-1.15) |
| Apgar score < 7 (5min) | 1.23 *** (1.18-1.28) | 1.17 *** (1.12-1.22) |
| Low birth weight | 1.13 *** (1.11-1.15) | 1.06 *** (1.04-1.09) |
| Small for gestational age | 1.04 *** (1.02-1.06) | 1.01 (0.99-1.03) |
| Cesarean section | 1.21 *** (1.20-1.22) | 1.16 *** (1.15-1.18) |
| All congenital malformations | 1.10 *** (1.04-1.17) | 1.07 * (1.01-1.14) |
| Nervous system | 1.19 (0.98-1.44) | 1.14 (0.93-1.39) |
| Eye-ear-face | 1.07 (0.94-1.21) | 1.04 (0.92-1.19) |
| Circulatory | 1.11 (0.95-1.29) | 1.05 (0.89-1.24) |
| Digestive | 1.20 (1.00-1.45) | 1.12 (0.92-1.37) |
| Urinary and genital | 1.04 (0.87-1.25) | 1.04 (0.86-1.26) |
| Musculoskeletal | 1.09 (0.97-1.23) | 1.06 (0.94-1.20) |
| Respiratory | 1.39 (0.99-1.95) | 1.21 (0.85-1.74) |
| Chromosomal abnormalities | 1.02 (0.88-1.19) | 1.02 (0.86-1.20) |
| Others | 1.06 (0.87-1.28) | 0.97 (0.79-1.19) |

**Note:** ^a^ Considering for mother’s age, child’s birth year, child’s sex, and mother’s comorbidities (hypertension, hyperlipidemia, diabetes mellitus and gestational diabetes mellitus); BZRA, benzodiazepine receptor agonist; IPTW, inverse probability of treatment weights**;** OR, odds ratio; CI, confidence interval; * P < 0.05; *** P < 0.001
