# Supplementary material for: Use of benzodiazepine receptor agonists in different pregnancy trimesters and risk of maternal and neonatal outcomes: a propensity weighted cohort study in Taiwan
Source: BMC Pregnancy Childbirth. 2025 Dec 6;25:1344. doi: 10.1186/s12884-025-08549-1 (PMC12751940; doi:10.1186/s12884-025-08549-1)
Supplement: Supplementary file 5 — Supplementary Material 5. [file 12884_2025_8549_MOESM5_ESM.docx]

Supplementary Table 5. Risk of adverse pregnancy outcomes between BZRA users and nonusers stratified by different comorbidities

| **Pregnancy outcomes** | **IPTW-OR (95% CI) ^a^** | | | | | |
| --- | --- | --- | --- | --- | --- | --- |
|  | **Anxiety** | | **Depression** | | **Insomnia** | |
|  | **Yes** | **No** | **Yes** | **No** | **Yes** | **No** |
| Stillbirth | 1.15 (0.89-1.50) | 1.19 *** (1.14-1.26) | 1.20 (0.83-1.74) | 1.19 *** (1.14-1.25) | 1.20 (1.00-1.45) | 1.19 *** (1.13-1.25) |
| Preterm | 1.16 ** (1.05-1.27) | 1.11 *** (1.09-1.13) | 1.36 *** (1.19-1.56) | 1.11 *** (1.09-1.13) | 1.21 *** (1.13-1.29) | 1.10 *** (1.08-1.13) |
| Apgar score < 7 | 1.03 (0.83-1.29) | 1.17 *** (1.13-1.22) | 1.05 (0.77-1.44) | 1.17 *** (1.12-1.22) | 1.10 (0.94-1.29) | 1.17 *** (1.13-1.23) |
| Low birth weight | 1.14 * (1.03-1.27) | 1.04 *** (1.02-1.06) | 1.23 ** (1.06-1.42) | 1.04 *** (1.02-1.06) | 1.14 ** (1.06-1.22) | 1.04 *** (1.02-1.06) |
| Small for gestational age | 1.01(0.92-1.11) | 1.00 (0.99-1.02) | 0.98 (0.86-1.12) | 1.00(0.99-1.02) | 1.01 (0.95-1.08) | 1.00 (0.99-1.02) |
| Cesarean section | 1.12 *** (1.05-1.18) | 1.16 *** (1.14-1.17) | 1.18 *** (1.08-1.28) | 1.15 *** (1.14-1.17) | 1.20 *** (1.15-1.26) | 1.15 *** (1.14-1.16) |
| All congenital malformations | 1.21 (0.87-1.67) | 1.06 (1.00-1.12) | 1.11 (0.70-1.78) | 1.06 (1.00-1.12) | 1.44 ** (1.16-1.78) | 1.04 (0.98-1.10) |

Note: ^a^ Considering for mother’s age, child’s birth year, child’s sex, and mother’s comorbidities (hypertension, hyperlipidemia, diabetes mellitus and gestational diabetes mellitus); IPTW, inverse probability of treatment weights**;** OR, odds ratio; CI, confidence interval; * P < 0.05; ** P < 0.01; *** P < 0.001
