# Supplementary material for: Use of benzodiazepine receptor agonists in different pregnancy trimesters and risk of maternal and neonatal outcomes: a propensity weighted cohort study in Taiwan
Source: BMC Pregnancy Childbirth. 2025 Dec 6;25:1344. doi: 10.1186/s12884-025-08549-1 (PMC12751940; doi:10.1186/s12884-025-08549-1)
Supplement: Supplementary file 6 — Supplementary Material 6. [file 12884_2025_8549_MOESM6_ESM.docx]

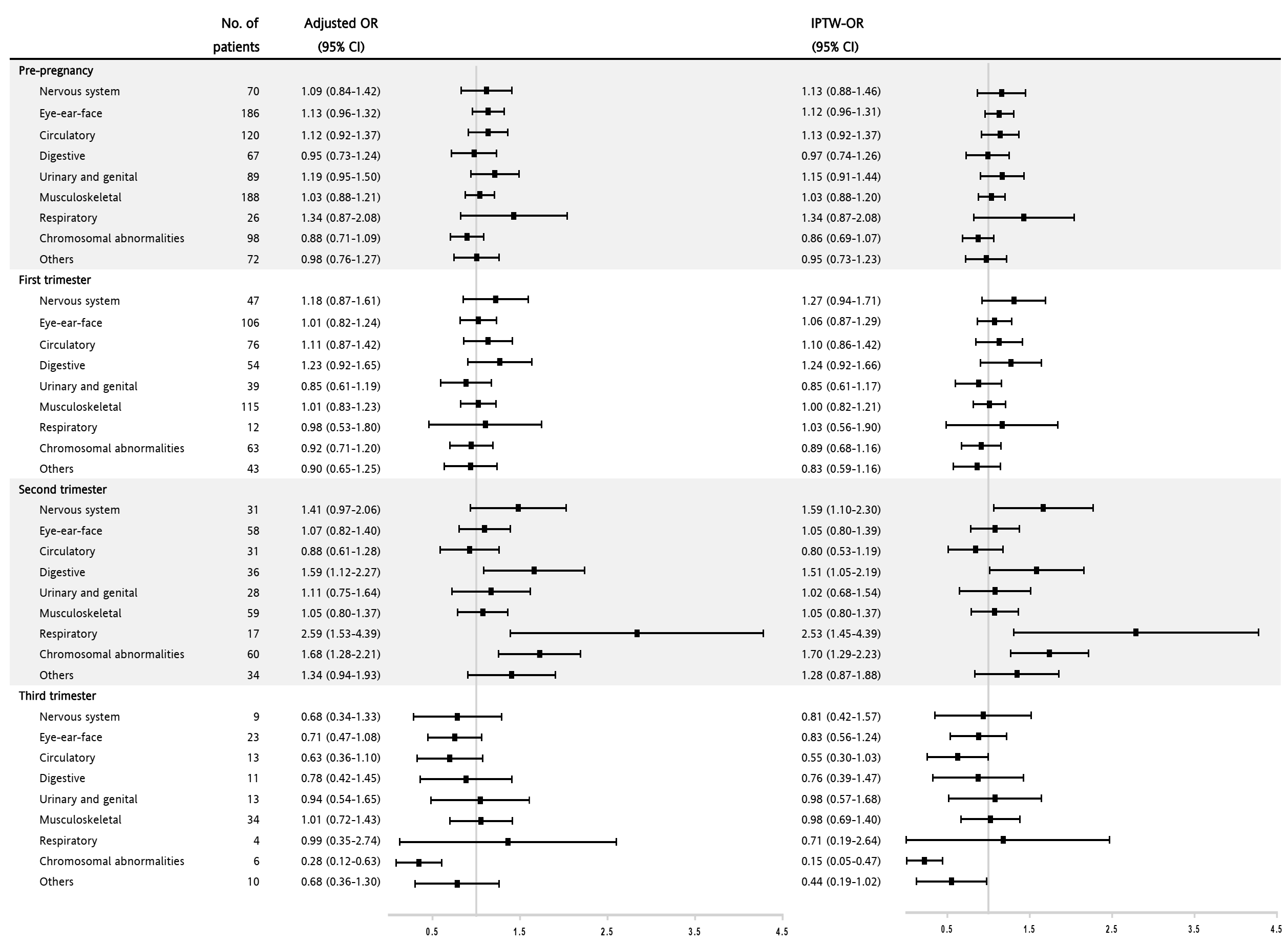


Supplementary Figure 1. Risk of different types of congenital malformations with maternal BZRA exposure in different trimesters

Note: Adjustment for mother’s age, child’s birth year, child’s sex, and mother’s comorbidities (hypertension, hyperlipidemia, diabetes mellitus and gestational diabetes mellitus); IPTW, inverse probability of treatment weights**;** OR, odds ratio; CI, confidence interval
